# Smoking cessation intervention during pregnancy in a Polish urban community – what is the target population?

**DOI:** 10.1186/1617-9625-1-9

**Published:** 2002-06-15

**Authors:** K Polańska, W Hanke, W Sobala, M Broszkiewicz

**Affiliations:** 1 Department of Environmental Epidemiology Nofer Institute of Occupational Medicine, Łódź, Poland; 2 Department of Social and Preventive Medicine Medical University, Łódź, Poland

## Abstract

The aim of this project was to evaluate the effect of intensive individual anti-smoking counselling among pregnant women from a Polish urban community with a large representation of socially underprivileged women. The study was conducted between 1 December 2000 and 31 December 2001. Out of 204 women who were asked to take part in a midwives-assisted program of educational counselling to stop smoking, 152 (74.5%) agreed to participate. The intervention program included four visits of a midwife trained in smoking cessation techniques to the home of a smoking pregnant woman. The control group were 145 pregnant women who on the first visit to a maternity unit received only a standard written information on the health risk from maternal smoking to the foetus. The percentage of pregnant women who quitted smoking during the project was 46.1% in the intervention group and 23.4% among the controls (p < 0.001). After combining the intervention group with the women who refused to participate in the project, the rate of quitting was 36.3%, still significantly higher than in controls (p = 0.01). The strongest influence of the intervention was found among women smoking more than 5 cigarettes/day. Women covered by the intervention programme, who reported smoking in previous pregnancies, were found to quit smoking to a much higher extent than the controls with a similar background. Such pattern was also observed for women whose husbands were smokers. The benefits of the intervention, especially for the socially underprivileged women, seem to result from an increased proportion of subjects who undertook a quitting attempt, rather than the effectiveness of these attempts. In the intervention group, among the subjects who did not manage to quit smoking during pregnancy, the number of women who at least slightly reduced their smoking rate was twice as high as in the controls.

## Introduction

From the late fifties, when the first report was published on the possible influence of maternal smoking on birth weight [1], hundreds of studies have demonstrated conclusively that smoking during pregnancy increases the risk of small-for-gestational-age infants and preterm delivery and, consequently, the perinatal mortality [2-4]. It was also observed that smokers who had changed their habit during pregnancy had a lower risk of these pathologies. A strong dose-response relationship has been found between the rate of smoking and the birth weight of their children [5,6]. More recent investigations imply that the health consequences of low birth weight can be seen in adult life e.g. as an elevated risk of cardiovascular diseases [7].

Butler and co-workers found that the infants of women who quitted smoking had the birth weights essentially the same as those of non-smokers [8]. However, the evidence that smoking cessation is effective has been provided by studies using the clinical trial design [9] often with an inclusion of objective biochemical measurements to confirm the smoking status of the examined groups [10-12]. A meta-analysis of the controlled trials of smoking cessation interventions during pregnancy concluded that overall there was a significant increase in the smoking cessation rates [13]. The benefits of these interventions, measured by the differences in the rate of quitters and average infant birth weight in the intervention and control populations, were varying and it was postulated that only the cognitive behavioural strategies are really effective [14].

The relative gain of the smoking cessation interventions depends not only on the type of activities undertaken but also on the characteristics of pregnant women. Despite the fact that the recognition of smoking as a hazard to reproductive health is fairly universal, one may hypothesize that for some groups of women it will be more difficult to quit smoking during pregnancy than for others. The decision-makers who are in charge of preventive health activities would like to be convinced that investment in the intervention programs in those specific target groups is really cost-effective.

What are these groups? There are only a few reports on the results of smoking cessation programs that would consider the social characteristics of smoking pregnant women [15,16]. This paper reports the results of a randomised controlled trial measuring the effect of intensive individual anti-smoking counselling in the population of Polish women from an urban community, with a large representation of socially underprivileged women.

## Methods

### Study populations

The cluster randomised trial was undertaken in the public district maternity centres in Łódź, Poland. The null hypothesis being tested was that the smoking cessation program conducted by midwives in the homes of pregnant women does not affect the quitting rate

Basing on data for 1999, we found that 15 maternity units in the Łódź district provide prenatal care for about 1600 pregnant women a year. It was estimated from previous surveys that the smoking rate in I trimester of pregnancy is about 30%. Thus, it could be expected that annually approx. 32 smoking pregnant women book for prenatal care in each maternity unit. We calculated that in order to find a significant difference between the quitting rates of 35% and 20%, assuming α = 0.05 and 80% power, the compared populations should include at least 137 subjects. Assuming the 30% refusal rate in the intervention group and about 5% among the controls, the former group should comprise at least 200 subjects and the latter one 144. We found that to accumulate populations of this size, six maternity units should participate in the intervention and four in the control procedures.

204 women in total were asked to take part in a midwives-assisted program of intensive educational counselling to stop smoking. Out of them, 152 (74.5%) agreed to participate in such activities. At the same period of time, 145 (100%) smoking pregnant women who booked for maternity care in four control units agreed to respond to an inquiry about their smoking status and to update this data in 20th month of pregnancy and after delivery.

The study was conducted between 1 December 2000 and 31 December 2001. We decided to use as controls a female population from a different administrative part of the city rather than to randomly allocate women to the intervention and control group from the same maternity units. We expected that the women participating in the program might have some influence on the latter group.

### Description of the intervention and control activities

On their first visit to one of six public maternity units participating in the smoking cessation program, all smoking pregnant women were informed by their physicians about the programme and asked whether they wanted to join it. According to the study protocol, a midwife trained in smoking cessation techniques was to visit the smoking pregnant women in their homes. The women received written materials prepared by Community Health Research Unit in Ottawa that were translated and adapted to the Polish conditions [17].

The programme included four midwife visits. The first visit included a diagnosis of the level of smoking addiction, using the Fagerström method, then discussing the benefits of smoking cessation, and encouraging the woman to take up a decision to quit smoking. The Fagerström Test for Nicotine Dependence measures physical dependence on nicotine [18]. This test includes 6 questions about the smoking habits. The total score above 6 points indicated that the woman examined may have been physically dependent on nicotine.

On the second visit, about 1–2 weeks later, the pregnant woman who decided to give up smoking determined when this was to be done and signed the "Declaration to quit smoking". On the third visit, scheduled 1–2 days after the expected quitting day, the midwife inquired whether the woman actually acted as she promised. On the fourth visit, one month after the quitting day, the midwife informed the woman how to avoid smoking and keep smoking abstinence. During all the four visits it was stressed that the program is supposed to provide support only to those women who attempt to change their smoking status. The counselling emphasised the potential benefits to the foetus but the decision to quit or not was left to the pregnant woman herself. Once she made a step in this direction, all her further efforts were repeatedly reinforced.

When the woman covered by the program did not manage to quit smoking during the four midwife's visits, she was offered a possibility to continue the intervention activities during another five visits.

Women from the control group, on the first visit to a maternity unit, received a standard written information about the health risk from maternal smoking to the foetus. At the same time, the physician who passed this information encouraged them to stop smoking.

### Scope of information obtained

During the initial contact with the maternity unit the subjects filled in the questionnaire that contained information about their smoking profile (number of cigarettes smoked, years of smoking, partner's smoking, other household members' smoking, smoking in previous pregnancies if any, previous smoking cessation attempts). Such information was obtained from all women participating in the smoking cessation program. Similar data were also collected from those who refused to participate and the control group.

In the control group, the data on smoking habit were updated in the 20th month of pregnancy whereas in the intervention group any changes in the smoking profile were recorded during each visit.

As regards the group of women who refused to participate, it was possible to elicit information about their social characteristics and smoking habit. These were collected during their first visit to maternity unit.

A few days after the delivery the midwives visited the women both from the intervention and control group in their homes. As for the former, they inquired whether anything had changed in their smoking status since the last meeting (maintaining abstinence, smoking relapses, quitting after the period of intervention). In the control group, the smoking status during the whole pregnancy was examined retrospectively.

### Data analysis

The data were analysed with the use of the SPSS package. The statistical measures of relationship included the t-test and the chi-square test or Fisher exact test as appropriate. The level of significance for acceptance of relationship between the variables was the conventional 0.05.

## Results

### Comparison of social characteristics of the examined groups

The intervention and control subjects had comparable demographic profiles (Table 1). The average age of women in both groups was 25.8 years. The intervention group was more frequently found to be unmarried and primigravida and to have only the primary or vocational education. None of these differences was statistically significant. No differences were noted between the two groups with respect to the employment status.

**Table 1:** Social characteristics of the intervention, control and refusal groups

Variable	Intervention (N = 152)		Control (N = 145)		Refusal (N = 52)	
	
	n	%	n	%	n	%
Education						
Primary or vocational	125	82.2	110	75.9	40	76.9
(8 or 11 years of education)						
College or university	27	17.8	35	24.1	12	23.1
(12 or 17 years of education)						
Marital status						
Married	73	48.0	86	59.3	18	34.6
Unmarried	79	52.0	59	40.7	34	65.4
Number of children						
0	81	53.3	61	42.1	29	55.8
1	35	23.0	41	28.3	12	23.1
≥ 2	36	23.7	43	29.6	11	21.1
Employment status						
Employed	55	36.2	51	35.2	19	36.5
Unemployed	97	63.8	94	64.8	33	63.5
Years of smoking						
<5	45	29.6	45	31.0	19	36.5
5–10	73	48.0	79	54.5	21	40.4
>10	34	22.4	21	14.5	12	23.1
No of cigarettes smoked/day						
<5	10	6.6*	13	9.0	4	7.7
5–10	65	42.8	82	56.5	27	51.9
>10	77	50.6	50	34.5	21	40.4
Fagerström test						
0–6	138	90.8*	143	98.6	46	88.5
7–9	14	9.2	2	1.4	6	11.5
Husband smoking						
Yes	130	85.5	111	76.6	46	88.5
No	22	14.5	34	23.4	6	11.5
Any other household member smoking?						
Yes	89	58.6*	63	43.4	33	63.5
No	63	41.4	82	56.6	19	36.5
Smoking in previous pregnancies						
Primigravidas	76	-	58	-	25	-
Yes	68	89.5	74	85.1	23	85.2
No	8	10.5	13	14.9	4	14.8

* statistically significant (p < 0.05) difference in distributions of groups intervention and control.

In the intervention group, the husbands/partners of the subjects were found to be smokers as often (85.5%) as those of the controls (76.6, p = 0.05). More pronounced differences were noted when other smokers in the household were taken into account (58.6% vs. 43.4%, p = 0.009). The women from the intervention group who were pregnant in the past, admitted smoking in previous pregnancies slightly more frequently than the controls did (89.5 vs. 85.1%, p = 0.4).

The intervention group smoked more cigarettes per day on average (12.3 ± 5.9 vs. 10.9 ± 5.7, p = 0.04). The level of smoking addiction measured by the Fagerström test was significantly higher in the intervention group (3.6 ± 2.1 vs. 2.2 ± 1.8, p < 0.001).

There were no statistically significant differences between the intervention and refusal groups. Women from the refusal group were more often unmarried than those from the intervention group (65.4 vs. 52.0, p = 0.09). In this group, the average period of smoking was 7.4 ± 5.9 and the number of cigarettes/day 12.3 ± 6.7.

### Rate of quitting smoking

The percentage of pregnant women who quitted smoking was 46.1% in the intervention group and 23.4% among the controls (p < 0.001) (Table 2). In the refusal group (N = 52) four women quitted smoking, while the smoking status of others was unknown. After combining the intervention group with the group of women who refused to participate in the project, the rate of quitting was 36.3%, thus still significantly higher (p = 0.01) than in the control group.

**Table 2:** Quitting smoking and social characteristics of the examined subjects (% calculated as a fractions of all subjects in the given subgroup)

Variable	Intervention			Control			Intervention and Refusal		
	
	N	n	%	N	n	%	N	n	%
All groups	152	70	46.1*	145	34	23.4	204	74	36.3**
Education									
Primary or vocational	125	52	41.6*	110	19	17.3	165	54	32.7**
College or university	27	18	66.7	35	15	42.9	39	20	51.3
Number of cigarettes/day smoked before the attempt									
<5	10	9	90.0*	13	5	38.5	14	9	64.3
5–10	65	35	53.8*	82	22	26.8	92	36	39.1
>10	77	26	33.8*	50	7	14.0	98	29	29.6**
Fagerström test									
0–6	138	63	45.7*	143	34	23.8	184	67	36.4**
7–9	14	7	50.0	2	0	0	20	7	35.0
Smoking in previous pregnancies									
Primigravidas	76	43	56.6*	58	20	34.5	101	45	44.6
Yes	68	20	29.4*	74	7	9.5	91	21	23.1**
No	8	7	87.5	13	7	53.8	12	8	66.7
Smoking husband									
Yes	130	58	44.6*	111	20	18.0	176	61	34.7**
No	22	12	54.5	34	14	41.2	28	13	46.4

* statistically significant (p < 0.05) difference in distributions of groups intervention and control;

** statistically significant (p < 0.05) difference in distributions of groups intervention and refusal combined and control.

The effect of the intervention was much more evident in the group with only primary or vocational education (41.6% vs. 17.3%, p < 0.001) than with a college or university degree (66.7% vs. 42.9%, p = 0.06). A similar pattern was observed in the combined intervention and refusal groups versus the control group.

When the number of cigarettes/day reported on the first visit was considered, the strongest influence of the intervention was found in the group smoking 5–10 cigarettes/day (53.8% vs. 26.8%, p < 0.001) and more than 10 cigarettes/day (33.8% vs. 14.0%, p = 0.01). Out of the subjects from the intervention group and the combined intervention and refusal group who scored above six on the Fagerström test, respectively fifty percent and thirty-five percent were found to quit smoking. Both findings were significantly higher than those obtained in the control group with similar test results.

The women covered by the intervention program who reported smoking in previous pregnancies were noted to quit smoking to a much higher extent than the controls with a similar background (29.4% vs. 9.5%, p = 0.002). A similar pattern was observed in the combined intervention and refusal groups versus the control group (23.1% vs. 9.5%, p = 0.02). Such pattern was also observed in the group of women whose husbands were smokers (44.6% vs. 18.0%, p < 0.001). After combining the intervention group with the group of women who refused to participate in the project, the rate of quitting was still significantly higher than in the control group (34.7 vs. 18.0, p = 0.02).

### Attempts to quit smoking

The intervention group almost twice as often as the controls undertook at least one attempt to quit smoking (78.9% vs. 40.0%, p < 0.001) (Table 3). This pattern could best be seen among the subjects with primary or vocational education. In the intervention group with such level of education 76.0% made such challenge, compared to 31.8% from the control group (p < 0.001). This finding was to a much lesser extent observed among women who had a college or university degree. It is worth noting that women from the intervention group who had primary or vocational education were trying to quit smoking even harder than those with a college or university degree in the control group (76.0% vs. 65.7%). As regards the percentage of women who had undertaken at least one quitting attempt, significant differences between the intervention and control subjects were found only in the subgroups smoking five or more cigarettes/day.

**Table 3:** Quitting attempts among the intervention and control subjects (% calculated as a fraction of all subjects in the given subgroup)

Variable	Intervention			Control		
	N	n	%	N	n	%

All groups	152	120	78.9*	145	58	40.0
Education						
Primary or vocational	125	95	76.0*	110	35	31.8
College or university	27	25	92.6*	35	23	65.7
Number of cigarettes/day smoked before the attempt						
< 5	10	10	100	13	11	84.6
5–10	65	53	81.5*	82	35	42.7
>10	77	57	74.0*	50	12	24.0
Fagerström test						
0–6	138	111	80.4*	143	58	40.6
7–9	14	9	64.3	2	0	0
Smoking in previous pregnancies						
Primigravidas	76	67	88.2*	58	31	53.4
Yes	68	45	66.2*	74	19	25.7
No	8	8	100	13	8	61.5
Smoking husband						
Yes	130	100	76.9*	111	38	34.2
No	22	20	90.9*	34	20	58.8

* statistically significant (p < 0.05) difference in distributions of groups intervention and control.

Sixty four percent of the intervention subjects who scored above six on the Fagerström test, compared to none of two controls with similar test results, undertook an attempt to quit smoking (p = 0.2).

The percentage of subjects from the intervention group who smoked in previous pregnancies and undertook a quitting attempt during the project was twice as large as that of the controls (66.2% vs. 25.7%, p < 0.001). A similar trend could be seen for the variable of living with smoking husband/partner (76.9% vs. 34.2%, p < 0.001).

### Effectiveness of smoking cessation attempts

The effectiveness of smoking cessation attempts (success rate), measured by the ratio of successful quitters to all who attempted to quit smoking, was 58.3% and 58.6%, respectively in the intervention and control groups (p = 1.0) (Table 4). No differences were found either in the success rate calculated for education subgroups. With respect to the number of cigarettes smoked, the effectiveness of quitting attempts was found to decrease with an increasing number of cigarettes/day in the intervention group. An opposite trend was observed in the control group. However, in view of the small number of subjects in each subgroup, the statistical significance of these patterns was not proved. Neither the results of the Fagerström test, nor the smoking in previous pregnancies or living with a non-smoking husband/partner enhanced the effectiveness of the quitting attempts.

**Table 4:** Effectiveness of quitting attempts by social variables (% calculated as a fraction of all subjects with quitting attempts in given subgroup)

Variable	Intervention			Control		
	N	n	%	N	n	%

All groups	120	70	58.3	58	34	58.6
Education						
Primary or vocational	95	52	54.7	35	19	54.3
College or university	25	18	72.0	23	15	65.2
Number of cigarettes/day smoked before the attempt						
< 5	10	9	90.0	11	5	45.5
5–10	53	35	66.0	35	22	62.9
>10	57	26	45.6	12	7	58.3
Fagerström test						
0–4	111	63	56.8	58	34	58.6
7–9	9	7	77.8	0	0	-
Smoking in previous pregnancies						
Primigravidas	67	43	64.2	31	20	64.5
Yes	45	20	44.4	19	7	36.8
No	8	7	87.5	8	7	87.5
Smoking husband						
Yes	100	58	58.0	38	20	52.6
No	20	12	60.0	20	14	70.0

### Smoking reduction among non-quitters

The two groups examined were compared with respect to the smoking reduction rate in the subgroups of women who did not manage to quit smoking during pregnancy (Table 5). In the intervention group, the number of subjects who were able to at least slightly reduce their smoking rate was twice as large as in the controls (91.5% vs. 50.5%, p < 0.001). Both groups reduced the number of cigarettes smoked by more than 50%, which corresponded to about 7–8 cigarettes/day on average.

**Table 5:** Changes in the smoking profile in the intervention and control groups (quitters excluded)

Examined group	Reduced number of cigarettes		No change		Mean daily reduction in cigarette consumption		Mean difference in the daily consumption of cigarettes	
	
	n	%	N	%	Av. %	SD	Av.	SD
Intervention (n = 82)	75	91.5	7	8.5	60.8	16.0	8.5	4.1
Control (n = 111)	56	50.5	55	49.5	57.1	14.5	7.5	3.9
p value	<0.001				0.1		0.09	

## Discussion

We found that the midwife-assisted smoking cessation intervention was effective when compared with the results obtained for controls covered by routine procedures. The quitting effect referred mainly to the subjects with primary education and who smoked more than 5 cigarettes/day at booking to the maternity unit.

The rates of quitting smoking we noted were much higher than those reported by other investigators [3,9,19-24]. The latter were usually at the level of 2–17% in the control group and 6–27% in the intervention one. The high percentage of quitters in the Polish population may be explained by the fact that in this country the population of smoking women to a much higher extent includes the occasional smokers who may have less problems with quitting smoking during pregnancy than those strongly addicted to tobacco. The quitting rate of 23,4% in the control group is very close to those reported in other Polish surveys of pregnant women [25,26]. The large proportion of quitters in the intervention group may result from a rather low participation rate of women to whom the intervention was offered. We may speculate that women who agreed to take part in the programme were more inclined to quit smoking than those who refused. No such selection bias was found in the case of controls.

We did not verify the self-reported smoking status by using biomarkers of exposure to tobacco smoke. From our previous investigations we know that about 20% of smokers may not admit that they smoke [26]. It may be possible that a similar proportion of smokers was not identified in our study and consequently was not assigned either to the intervention or control group. We cannot predict whether the inclusion of representatives of this group would result in an increase in the quitting rate (as they are more conscious of the smoking hazard) or a decrease (as they are not willing to accept any counselling). Our position is that whatever the direction of this bias might be, it should refer to both the groups compared, hence the final findings should not be affected.

One of the consequences of not using any validation of the smoking status may be an overestimation of the quitting rate in the intervention group, since they may have reported false results just to please their counsellors. However, as a consequence, the effectiveness of such attempts must be lower in the intervention group (those claiming false attempts would not report abstinence for a long period of time and their attempt would be registered as unsuccessful), which was not the case in our study.

Almost all the non-quitters in the intervention group and half of them in the controls reported some reduction in the amount of cigarettes smoked. These rates are twice as high as in the study of Hartman et al. [10] in which resident physicians provided self-helped materials to intervention subjects. The explanation may be what was already mentioned that the smoking pregnant women in Poland derive mainly from the population of occasional smokers, and those of them who do not manage to quit smoking can at least reduce the number of cigarettes smoked during pregnancy. It is also possible that the women willing to stop smoking may have been over-represented in the intervention group.

Both the maternity units participating in the intervention program and the control ones were selected randomly. However, due to a substantial rate of non-participants the two examined groups were not exactly comparable. To eliminate the possible bias resulting from this situation, two major variables were controlled that affected the decision to cease smoking i.e. education and cigarette consumption prior to intervention. A similar approach was used for other characteristics well known to hinder the quitting attempts: smoking husbands/partners, other smoking household member, smoking in previous pregnancies.

## Conclusion

The midwife-assisted smoking cessation intervention seems to be an effective tool in the activities to help pregnant smokers from urban communities, where the smoking rates are relatively high, to make a decision to quit smoking in view of the potential hazard to their pregnancies and the children to be born. This kind of preventive program is well-suited for women with low education, who are heavy smokers and who do not receive support from their families while making efforts to give up smoking. More information about the potential benefits of the proposed intervention will be gathered when the analysis of pregnancy outcomes has been completed.

## Competing interests

The authors declare that they have no competing interests.
